# The complete chloroplast genome sequence of the medicinal plant *Sophora tonkinensis*

**DOI:** 10.1038/s41598-020-69549-z

**Published:** 2020-07-27

**Authors:** Fan Wei, Danfeng Tang, Kunhua Wei, Fang Qin, Linxuan Li, Yang Lin, Yanxia Zhu, Aziz Khan, Muhammad Haneef Kashif, Jianhua Miao

**Affiliations:** 1 Guangxi Key Laboratory of Medicinal Resources Protection and Genetic Improvement, Guangxi Botanical Garden of Medicinal Plants, Nanning, 530023 Guangxi China; 2 Key Laboratory of Plant Genetics and Breeding, College of Agriculture, Guangxi University, Nanning, 530005 Guangxi China

**Keywords:** DNA sequencing, Plant evolution

## Abstract

*Sophora tonkinensis* belongs to genus *Sophora* of the Fabaceae family. It is mainly distributed in the ridge and peak regions of limestone areas in western China and has high medicinal value and important ecological functions. Wild populations of *S. tonkinensis* are in danger and need urgent conservation. Furthermore, wild *S. tonkinensis* resources are very limited relative to the needs of the market, and many adulterants are present on the market. Therefore, a method for authenticating *S. tonkinensis* and its adulterants at the molecular level is needed. Chloroplast genomes are valuable sources of genetic markers for phylogenetic analyses, genetic diversity evaluation, and plant molecular identification. In this study, we report the complete chloroplast genome of *S. tonkinensis.* The circular complete chloroplast genome was 154,644 bp in length, containing an 85,810 bp long single-copy (LSC) region, an 18,321 bp short single-copy (SSC) region and two inverted repeat (IR) regions of 50,513 bp. The *S. tonkinensis* chloroplast genome comprised 129 genes, including 83 protein-coding genes, 38 transfer RNA (tRNA) genes, and 8 ribosomal RNA (rRNA) genes. The structure, gene order and guanine and cytosine (GC) content of the *S. tonkinensis* chloroplast genome were similar to those of the *Sophora alopecuroides* and *Sophora flavescens* chloroplast genomes*.* A total of 1,760 simple sequence repeats (SSRs) were identified in the chloroplast genome of *S. tonkinensis*, and most of them (93.1%) were mononucleotides. Moreover, the identified SSRs were mainly distributed in the LSC region, accounting for 60% of the total number of SSRs, while 316 (18%) and 383 (22%) were located in the SSC and IR regions, respectively. Only one complete copy of the *rpl2* gene was present at the LSC/IRB boundary, while another copy was absent from the IRA region because of the incomplete structure caused by IR region expansion and contraction. The phylogenetic analysis placed *S. tonkinensis* in Papilionoideae, sister to *S. flavescens*, and the genera *Sophora* and *Ammopiptanthus* were closely related. The complete genome sequencing and chloroplast genome comparative analysis of *S. tonkinensis* and its closely related species presented in this paper will help formulate effective conservation and management strategies as well as molecular identification approaches for this important medicinal plant.

## Introduction

**Figure 1:**
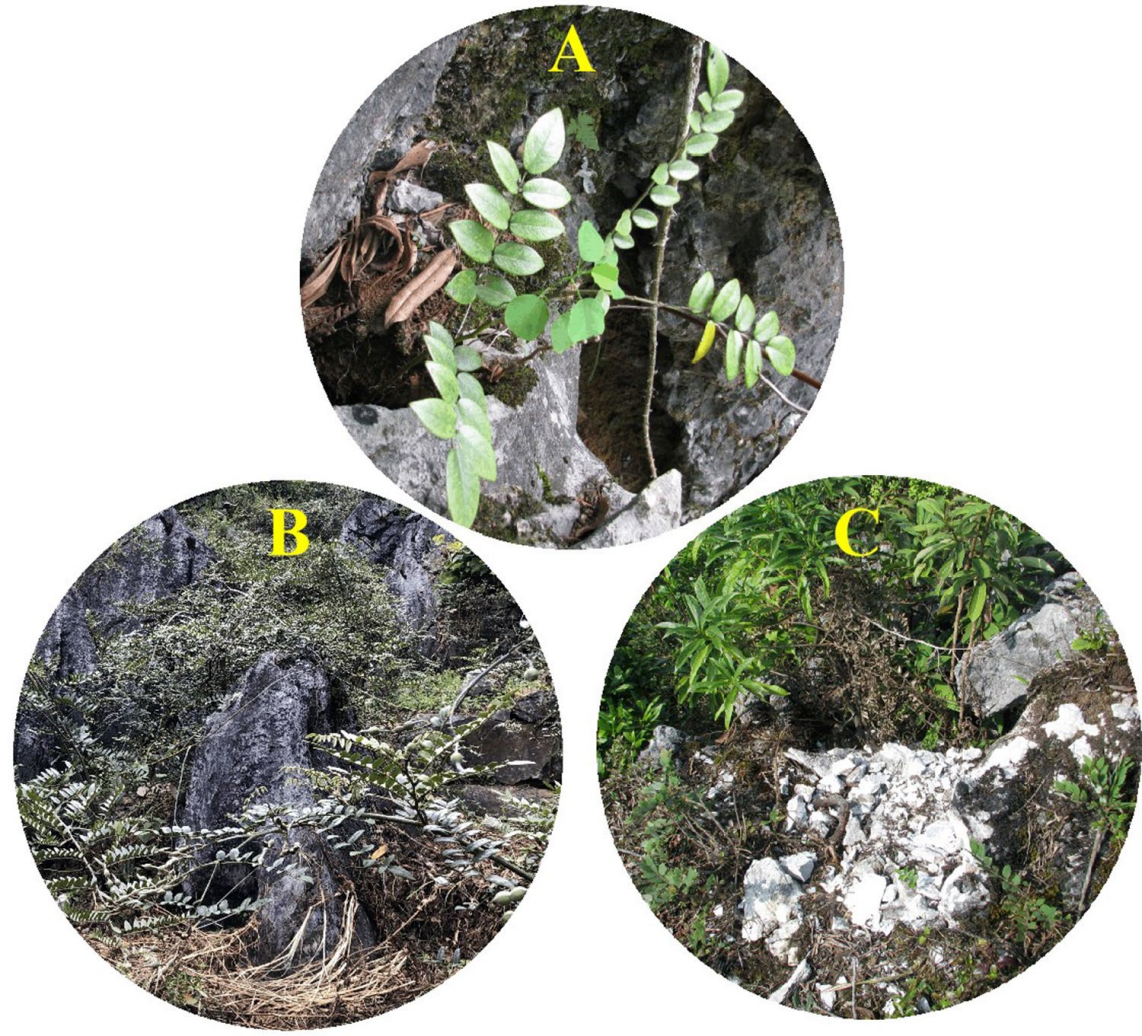
(**A**) The wild habitat of *S. tonkinensis.* (**B**) Habitat destruction of *S. tonkinensis.* (**C**) *S. tonkinensis* is used as an efficient species for the ecological restoration of karst rocky desertification areas.

*Sophora tonkinensis* is an important medicinal plant species in the genus *Sophora*, which belongs to Papilionoideae, a subfamily of the Fabaceae. This species is distributed mainly in Baise city, Jinchengjiang city, and Donglan County of Guangxi province as well as Guizhou and Yunnan Provinces of China^1^. *S. tonkinensis* grows mostly in ridge and peak regions of limestone areas in the wild and is used as an efficient species for the ecological restoration of karst rocky desertification areas (Fig. 1A,B)^2^. *S. tonkinensis* is effective in curing acute pharyngolaryngeal infection, eczema, colpitis, sore throat, gastrointestinal haemorrhage, and acute dysentery diseases^3^. The active ingredients in *S. tonkinensis* Gagnep mainly consist of alkaloids, saponins, flavonoids, and polysaccharides. Studies have shown that *S. tonkinensis* has various pharmacological effects, such as antitumour, anti-inflammatory, anti-arrhythmic, anti-diarrhoea, analgesic, and immune regulatory effects, as well as anti-hepatic fibrosis and liver-protective activities^4^.

Due to the unique habitat and extremely low reproductive capacity of *S. tonkinensis*, its wild resources are very limited relative to the needs of the market^5^. The species is under threat due to commercial overexploitation and serious habitat destruction (Fig. 1C), and its wild populations have been seriously shrinking. However, little is known regarding its genetic background. The plant chloroplast genome, with a length of 110–160 kb, is a valuable source of genetic markers for phylogenetic analyses, genetic diversity evaluation, and plant molecular identification due to its conserved structure and comparatively high substitution rate^6,7^. Therefore, a good understanding of chloroplast genomic information will make it easy to study genetic variation in and design reasonable conservation strategies for wild populations of *S. tonkinensis*.

Furthermore, there are many adulterants of *S. tonkinensis* on the market, and it is difficult to distinguish them according to outward appearance^8^, indicating an urgent need for a molecular approach with which to differentiate *S. tonkinensis* species from other adulterating species. DNA barcode sequence analysis, a molecular identification technology, can provide a rapid, accurate, and automatable method of species identification using a standardized piece of DNA sequence^9–11^. Chloroplast non-coding regions have been successfully applied in DNA barcoding research. Yao et al. found that the *psbA-trnH* intergenic spacer region could be used as a barcode to distinguish various *Dendrobium* species and to differentiate them from adulterating species^12^. Chen et al. tested the discrimination ability of *ITS2* in more than 6,600 plant samples belonging to 4,800 species from 753 distinct genera and found that the rate of successful identification with *ITS2* was 92.7% at the species level^13^. Chloroplast genomic information for *S. tonkinensis* will provide candidate DNA barcodes for the authentication of *S. tonkinensis* and the identification of its adulterants.

In the present study, we assembled and analysed the chloroplast genome sequence of *S. tonkinensis* based on Illumina paired-end (PE) sequencing data. The sequence was also compared with other known chloroplast genome sequences using bioinformatics analysis, and the evolutionary position of *S. tonkinensis* among the Papilionoideae was confirmed.

## Results

### Genome sequencing and assembly

In this study, PE DNA sequencing was carried out using the Illumina MiSeq sequencing platform. In total, 17,594,210*2 PE reads and 5,313,451,420 bases were obtained, and a nucleotide quality score greater than 20 (Q20) was achieved at a rate of 96.92%. After quality filtering, 16,892,769*2 PE reads, 663,584 single reads, and 5,058,544,355 bases were obtained. According to the total length of the assembled sequence, number of scaffolds and scaffold N50, the assembly results for multiple K-mers were evaluated comprehensively, and then the optimal-K-mer data were selected as the final assembly results. We obtained 1 scaffold with a length of 154,644 bp. These data demonstrated a high-quality assembly. The complete chloroplast genome sequence of *S. tonkinensis *was deposited in the Sequence Read Archive (SRA) (accession number: SRR8434290).

### General features of the *S. tonkinensis* chloroplast genome

**Figure 2:**
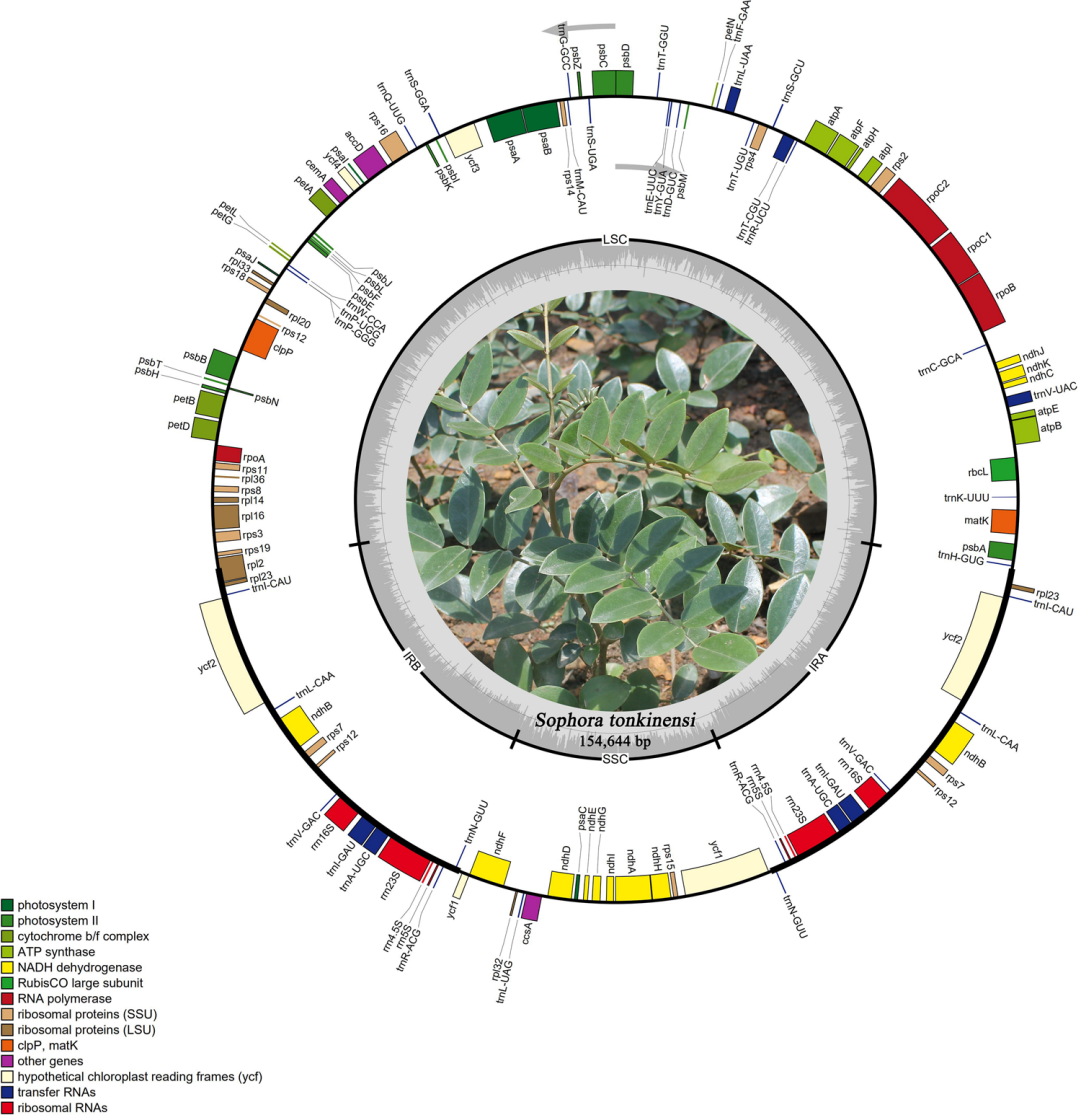
Chloroplast genome map of *S. tonkinensis.* Genes shown in the inner side of the circle are transcribed clockwise and those located on the outside of the circle are transcribed counter-clockwise. Genes belonging to different functional groups are colour-coded. Dashed area in the inner circle indicates the GC content of the chloroplast genome.

**Figure 3:**
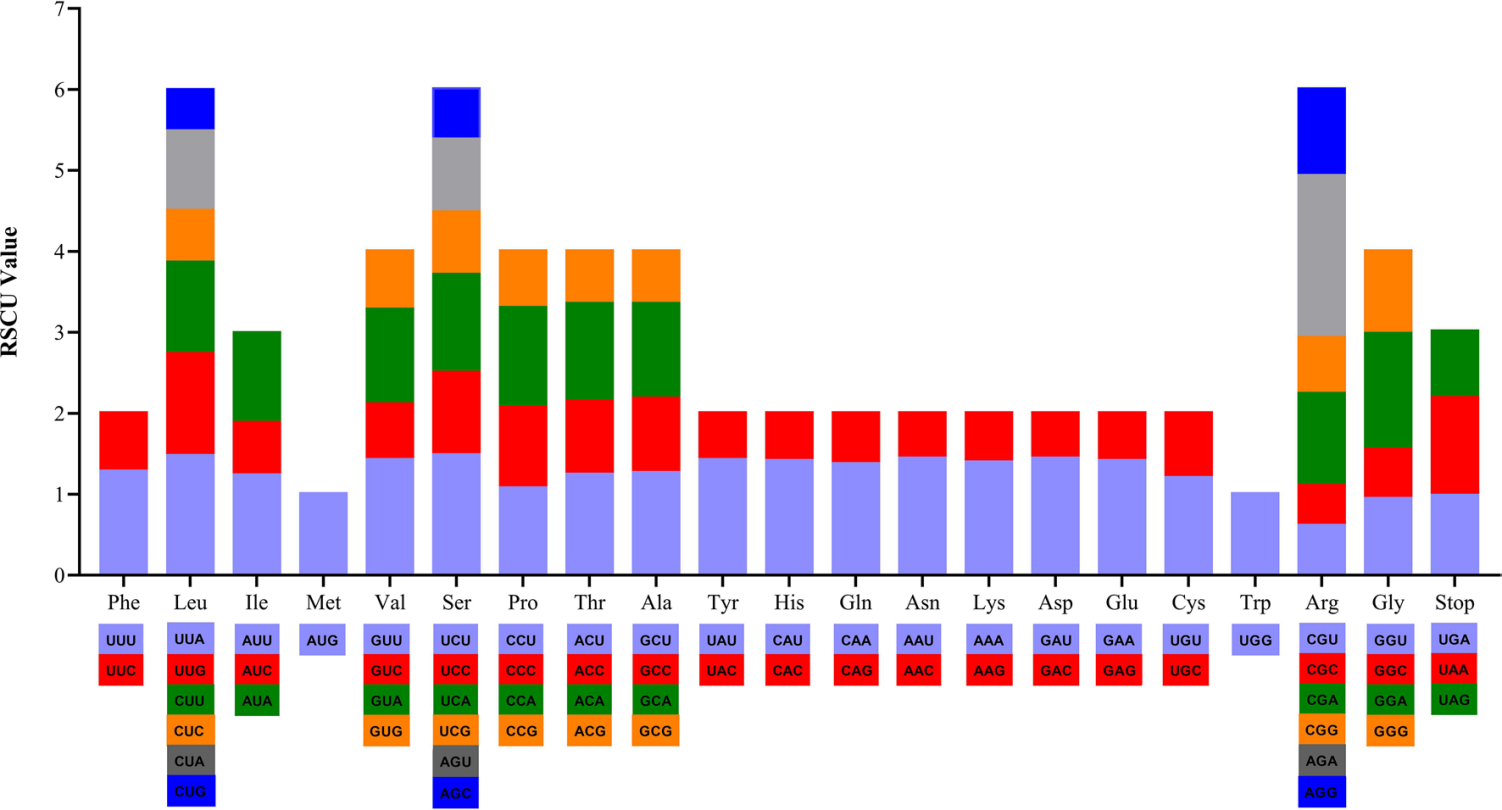
Relative synonymous codon usage (RSCU) of 20 amino acid and stop codons in all protein-coding genes of the chloroplast genome of *S. tonkinensis.*

Overall, the *S. tonkinensis* chloroplast genome was 154,644 bp in length and presented a complete circular structure, including a pair of inverted repeats (IRs) (50,513 bp) that divided the genome into two single-copy regions (long single-copy (LSC) region of 85,810 bp; short single-copy (SSC) region of 18,321 bp) (Fig. 2). Coding regions (60,756 bp) accounted for 39.3% of the genome, and intergenic regions (93,888 bp) comprised the remaining 60.7%. The percentages of guanine and cytosine bases (GC %) in the gene regions (37.8%) were higher than those in the intergenic regions (35.4%). The average gene length and gene density were 732 bp and 0.484, respectively. The frequencies of the four bases adenine (A), thymine (T), cytosine (C), and guanine (G) in the *S. tonkinensis* chloroplast genome were 49,139, 49,198, 27,915, and 28,392, accounting for 31.7%, 31.8%, 18.1%, and 18.4% of the genome, respectively (Table 1). The chloroplast genome of *S. tonkinensis* contained a total of 129 genes, including 83 protein-coding genes, 38 transfer RNA (tRNA) genes, and 8 ribosomal RNA (rRNA) genes (Table 2). Of the 83 protein-coding genes, nine (*rps16*, *rpoC1*, *atpF*, *petB*, *petD*, *rpl16*, *rpl2*, *ndhA* and *ndhB*) contained one intron, while *clpP* and *ycf3* possessed two introns (Table S1). The *S. tonkinensis* chloroplast genome contained 64 types of codons encoding 21 types of amino acids (Fig. 3). The number of codons differed from 247 to 2,320, with a fraction ranging from 0.08 to 1. The amino acids Met and Trp had only one codon, while the remaining amino acids possessed 2–6 codons.

**Table 1:** Summary of chloroplast genome characteristics of *S. tonkinensis*.

Characteristics	Number
Total length (bp)	154,644
LSC length (bp)	85,810
SSC length (bp)	18,321
IR length (bp)	50,513
GC content (%)	36.41
Gene total length (bp)	60,756
Gene number	83
Genes duplicated in IR	6
Gene average length (bp)	732
Gene density (number/kb)	0.484
Gene/genome (%)	39.3
GC content in gene region (%)	37.8
Intergenetic region length (bp)	93,888
Intergenetic length/genome (%)	60.7
GC content in intergenetic region (%)	35.4
tRNA gene	38
rRNA gene	8
rRNA gene duplicated in IR	4
A (bp)	49,139
T (bp)	49,198
G (bp)	28,392
C (bp)	27,915

**Table 2:** List of protein-coding genes present in the *S. tonkinensis* chloroplast genome.

Category	Gene group	Gene name					
Genes for photosynthesis	Subunits of photosystem I	*psaA*	*psaB*	*psaC*	*psaI*	*psaJ*	
	Subunits of photosystem II	*psbA*	*psbB*	*psbC*	*psbD*	*psbE*	*psbF*
		*psbH*	*psbI*	*psbJ*	*psbK*	*psbL*	*psbM*
		*psbN*	*psbT*	*psbZ*			
	Subunits of ATP synthase	*atpA*	*atpB*	*atpE*	*atpF*^b^	*atpH*	*atpI*
	Subunits of cytochrome	*petA*	*petB*^b^	*petD*^b^	*petG*	*petL*	*petN*
	Large subunit of Rubisco	*rbcL*					
	Subunits of NADH dehydrogenase	*ndhA*^b^	*ndhB*^ab^^c^	*ndhC*	*ndhD*	*ndhE*	*ndhF*
		*ndhG*	*ndhH*	*ndhI*	*ndhJ*	*ndhG*	
Self-replication	Small subunit of ribosome	*rps2*	*rps3*	*rps4*	*rps7*^ac^	*rps8*	*rps11*
		*rps12*^ac^	*rps14*	*rps15*	*rps16*^b^	*rps18*	*rps19*
	Large subunit of ribosome	*rpl2*^a^^b^	*rpl14*	*rpl16*^b^	*rpl20*	*rpl23*^ac^	*rpl32*
		*rpl33*	*rpl36*				
	DNA-dependent RNA polymerase	*rpoA*	*rpoB*	*rpoC1*^b^	*rpoC2*		
Other genes	Maturase	*matK*					
	Envelope membrane protein	*cemA*					
	Subunit of acetyl-CoA	*accD*					
	C-type cytochrome synthesis gene	*ccsA*					
	Protease	*clpP*^b^					
Unknown	Conserved hypothetical chloroplast reading frames	*ycf1*^c^	*ycf2*^ac^	*ycf3*^b^	*ycf4*		

^a^Genes located in the IR regions.

^b^Genes having introns.

^c^Two gene copies in IRs.

### Comparison to the chloroplast genomes of other *Sophora* species

The size of the *S. tonkinensis* chloroplast genome was found to be similar to those of the *Sophora alopecuroides* and *Sophora flavescens* chloroplast genomes^14,15^ (Table 3). However, the *S. tonkinensis* chloroplast genome had the longest LSC region (85,809 bp), whereas the *S. alopecuroides* chloroplast genome had the shortest LSC region (84,221 bp). As shown in Table 3, *S. tonkinensis* had the lowest GC content (36.4%), while *S. alopecuroides* and *S. flavescens* had the same higher GC content (36.6%). A total of 129 genes and 8 rRNA genes (four rRNA species) were identified in every species. *S. tonkinensis* and *S. alopecuroides* contained 83 protein-coding genes, whereas *S. flavescens* contained 84. The *S. tonkinensis* and *S. alopecuroides* chloroplast genomes possessed 38 tRNA genes, whereas the *S. flavescens* chloroplast genome possessed 37.

**Table 3:** Comparison of general features of the genus *Sophora* chloroplast genomes.

Genome feature	*S. tonkinensis*	*S. alopecuroides*	*S. flavescens*
Total length (bp)	154,644	154,108	154,378
LSC length (bp)	85,809	84,221	84,516
SSC length (bp)	18,320	18,139	18,110
IR length (bp)	50,515	51,748	51,752
Total genes	129	129	130
Protein gene	83	83	84
tRNA gene	38	38	37
rRNA gene	8	8	8
GC content (%)	36.4	36.6	36.6

### Simple sequence repeat (SSR) and tandem repeat analyses

**Figure 4:**
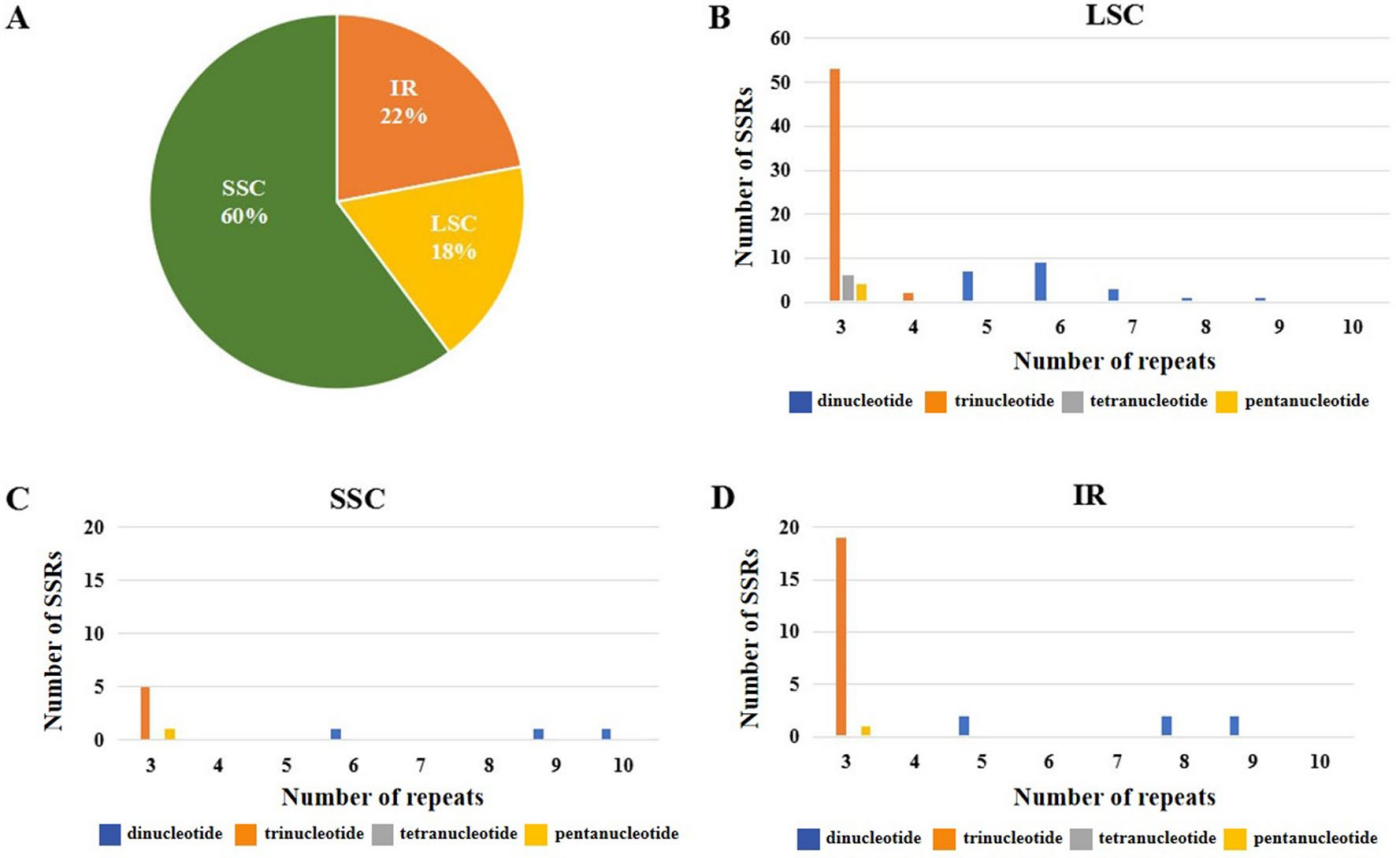
The distribution, type and presence of simple sequence repeats (SSRs) in the chloroplast genome of *S. tonkinensis*. (**A**) Presence of SSRs in the LSC, SSC, and IR regions. (**B**) Presence of polymers in the LSC regions. (**C**) Presence of polymers in the SSC regions. (**D**) Presence of polymers in the IR regions.

SSRs are molecular markers with high variation within the same species that are used in population genetic and polymorphism studies. The types, presence, and distribution of SSRs in the chloroplast genome of *S. tonkinensis* were studied. A total of 1,760 SSRs were identified, including 1,639 (93.1%) mononucleotide, 30 (1.7%) dinucleotide, 79 (4.5%) trinucleotide, 6 (0.3%) tetranucleotide, and 6 (0.3%) pentanucleotide repeats, and the mononucleotide A and T repeat units accounted for the largest portion, with a percentage of 81.9%. Moreover, the SSRs were mainly distributed in the LSC region, accounting for 60% of the total SSRs, while 316 (18%) and 383 (22%) were located in the SSC and IR regions, respectively (Fig. 4A). Of these, 21 dinucleotide, 55 trinucleotide, 6 tetranucleotide, and 4 pentanucleotide repeats were identified in the LSC region; 3 dinucleotide repeats, 5 trinucleotide repeats, and 1 pentanucleotide repeats were found in the SSC region; and 6 dinucleotide repeats, 19 trinucleotide repeats, and 1 pentanucleotide repeat were observed in the IR region (Fig. 4B–D). The size and location of the tetra- and pentapolymers are shown in Table S2. Of these repeats, 10 and 2 were localized in intergenic spacers and coding regions, respectively, and none were found in introns.

Tandem repeat sequences play a crucial role in genome rearrangement and phylogenetic analysis^16^. In the current study, a total of 23 tandem repeats were identified in the *S. tonkinensis* chloroplast genome (Table S3), which was smaller than the numbers observed in *S. alopecuroides* (49)*, Ammopiptanthus mongolicus* (39), and *Maackia floribunda* (64) of Papilionoideae . Most of the tandem repeats were distributed in intergenic spacers and introns (19 (82.7%) in the intergenic spacers and 1 in the intron of *clpP*), and just 2 and 1 were located in the protein-coding regions of *ycf2* and *ndhF*, respectively.

### Comparative analysis of the *S. tonkinensis* chloroplast genome

**Figure 5:**
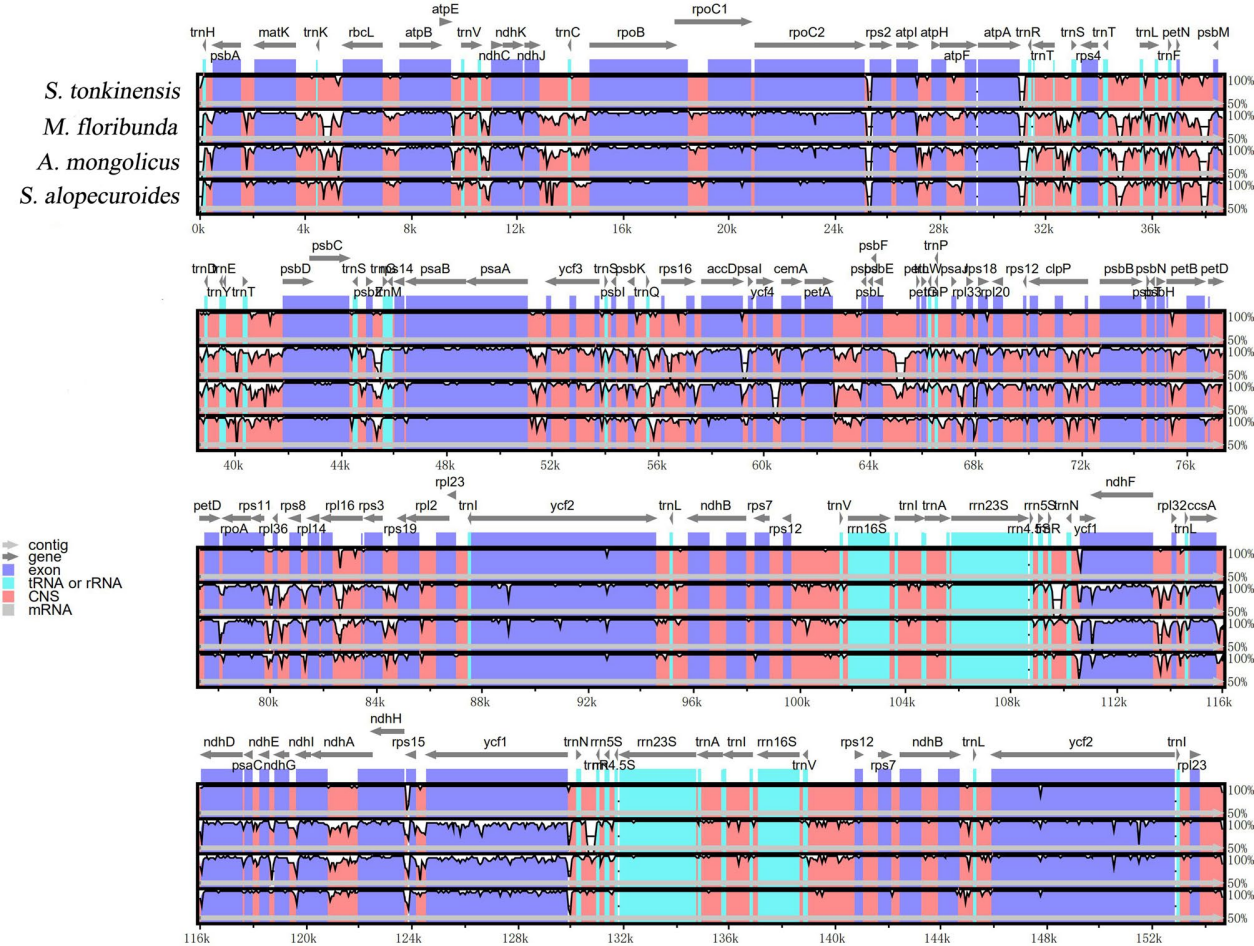
Comparison of the chloroplast genome sequences of *S. tonkinensis*, *S. alopecuroides*, *A. mongolicus,* and *M. floribunda* generated with mVISTA. Grey arrows indicate the position and direction of each gene. Red and blue areas indicate the intergenic and genic regions, respectively. The vertical scale indicates the percentage of identity, ranging from 50 to 100%.

Three published sequences representing *Sophora* (*S. alopecuroides*)*, Ammopiptanthus* (*A. mongolicus*), and *Maackia* (*M. floribunda*) of Papilionoideae were selected for comparison with the sequence of *S. tonkinensis* to estimate the sequence divergence of different regions of these plastomes. The overall sequence identities of the four Papilionoideae chloroplast genomes were plotted using mVISTA with the annotation of *S. tonkinensis* as the reference, and we observed approximately identical gene orders and organizations among them (Fig. 5). The coding regions were found to be more highly conserved than the non-coding regions, and the two IR regions were less divergent than the LSC and SSC regions. The most divergent coding regions of the four chloroplast genomes were *ycf1*, *ndhF*, *accD*, *rpoC2*, and *rpoB,* and the four rRNA genes (rrn4.5, rrn5, rrn16, and rrn23) were the most conserved.

**Figure 6:**
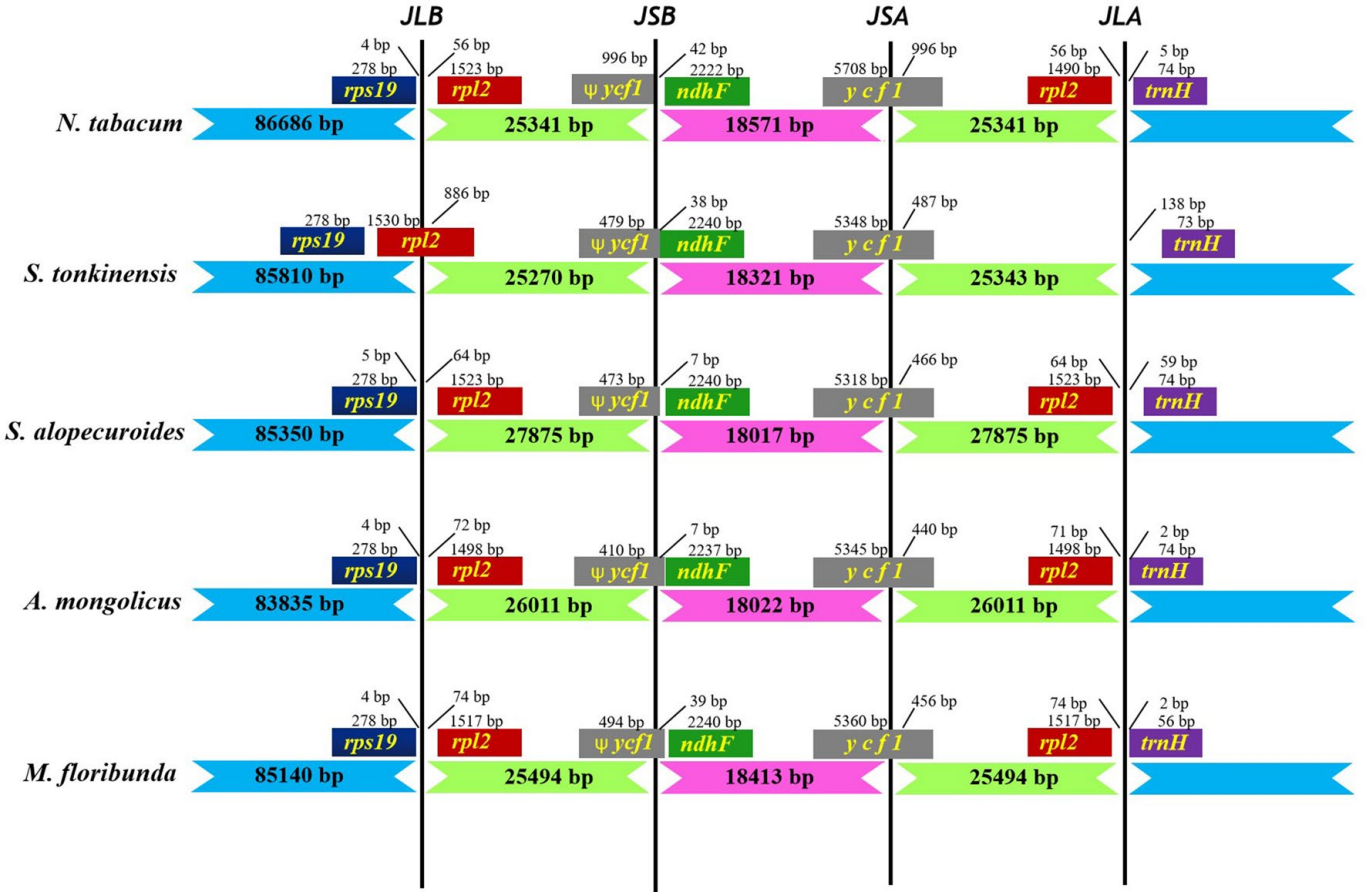
Comparison of the border regions among LSC, IR and SSC in the chloroplast genomes of *S. tonkinensis*, *S. alopecuroides*, *A. mongolicus,* and *M. floribunda.* JLB: junction line between LSC and IRb; JSB: junction line between IRb and SSC; JSA: junction line between SSC and IRa; JLA: junction line between IRa and LSC.

IRs are the most conserved regions in the chloroplast genome, and contraction and expansion at their boundaries are common evolutionary events, representing one of the main factors affecting chloroplast genome size. Using *Nicotiana tabacum* as the reference species, we compared the IR/LSC and IR/SSC borders of the chloroplast genomes of *S. tonkinensis, S. alopecuroides, A. mongolicus,* and *M. floribunda* of Papilionoideae (Fig. 6). The results showed that *S. tonkinensis* had size differences in the LSC, SSC and IR regions compared with those in other closely related chloroplast genomes of Papilionoideae species. In all of these species, the *rps19* gene was located in the LSC region. The *rpl2* gene of *S. tonkinensis* spanned the LSC and IRB regions, while the *rpl2* genes of the other species were all observed in the IRB region, with a 4–5 bp distance from the LSC/IRB border. The *ycf1* pseudogene spanned the IRB/SSC boundary in all chloroplast genes, while the *yfc1* pseudogene and *nadH* gene overlapped in *A. mongolicus*. The *nadH* gene was present in the SSC region of all genomes, with a 7–74 bp distance from the IRB/SSC junction. Expansion and contraction of the *ycf1* gene were observed in the boundary regions of the SSC/IRA. Size variation in *ycf1* from 5,318 to 5,708 bp was identified in all chloroplast genomes. The *trnH* gene was found in the LSC region of all genomes but was located 2 to 138 bp from the IRA/LSC boundary. In *S. tonkinensis*, the *rpl2* gene was absent in the IRA region because of the incomplete gene structure caused by the expansion and contraction of IR regions.

### Synonymous (K_S_) and non-synonymous (K_A_) substitution rate analysis

**Figure 7:**
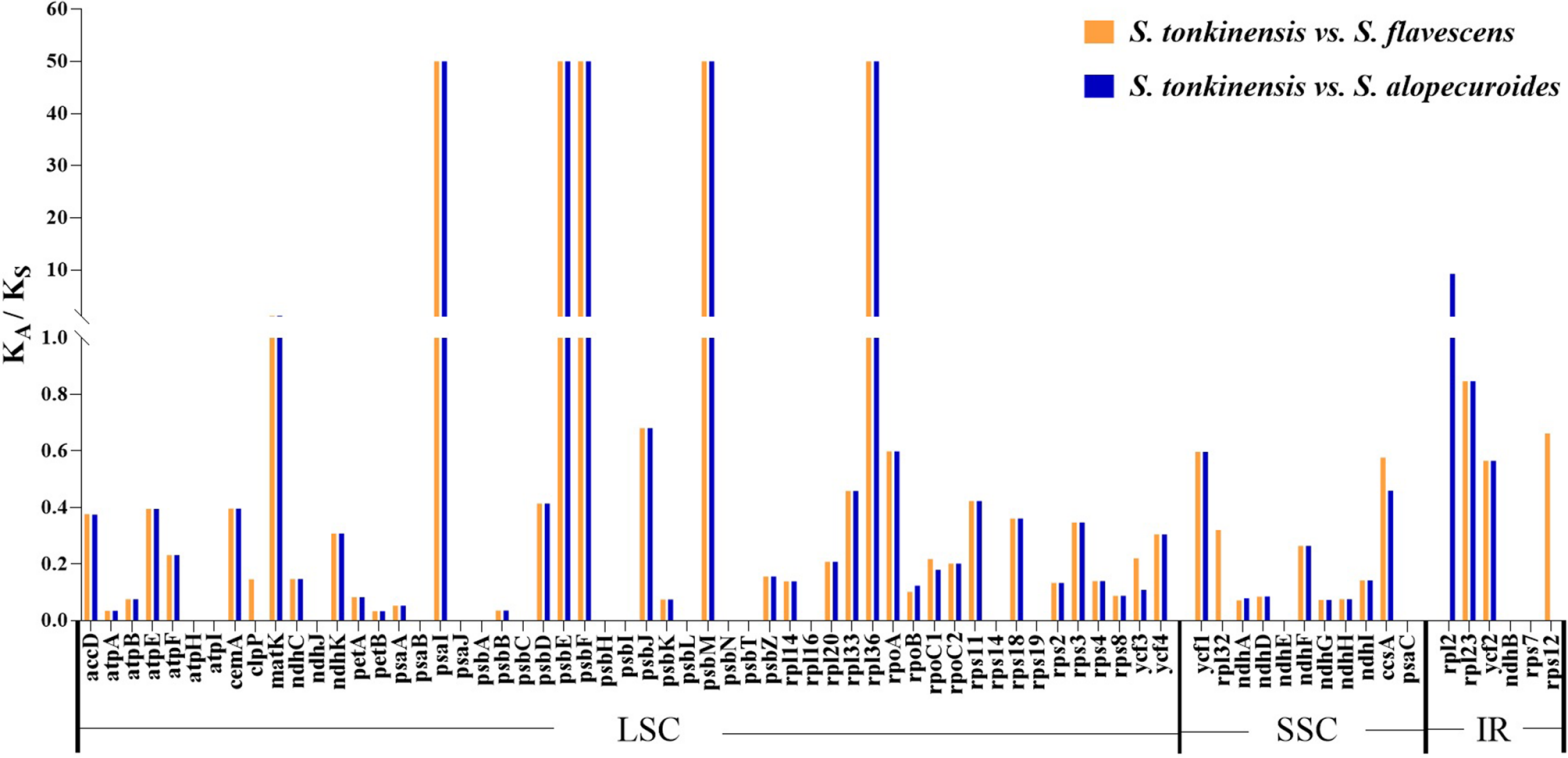
K_A_/K_S_ values of 70 protein-coding genes in the *S. tonkinensis Vs. S. alopecuroides* and *S. tonkinensis Vs. S. flavescens* comparisons*.* Orange coloured bars indicate *S. tonkinensis Vs S. flavescens*, and blue coloured bars indicate *S. tonkinensis Vs*. *S. alopecuroides.*

A total of 70 genes in the chloroplast genome of *S. tonkinensis* were used to calculate the K_A_/K_S_ ratio relative to the chloroplast genome of *S. alopecuroides* and *S. flavescens* (Fig. 7). The K_A_/K_S_ ratios of most of the genes in *S. tonkinensis Vs.* those in *S. flavescens* and *S. alopecuroides* were consistent with negative (or purifying) selection (K_A_/K_S_ < 1), while six genes (*matK*, *psbE*, *psbF*, *psbM*, *psaI*, and *rpl36*) displayed positive selection (K_A_/K_S_ > 1). Notably, the K_A_/K_S_ ratios of *psbE*, *psbF*, *psbM*, *psaI*, and *rpl36* in the *S. tonkinensis Vs. S. flavescens* and *S. alopecuroides* comparisons were as high as 50, which indicated great evolutionary divergence in these genes. The *rps2* and *rpl32* genes were differentially selected: *rps12* did not differ in the *S. tonkinensis Vs. S. flavescens* comparison*,* but it was positively selected in the *S. tonkinensis Vs. S. alopecuroides* comparison (K_A_/K_S_ = 9.25). *rpl32* exhibited no difference in the *S. tonkinensis Vs. S. alopecuroides* comparison but was negatively selected in the *S. tonkinensis Vs. S. flavescens* (K_A_/K_S_ = 0.32) comparison.

### Single nucleotide polymorphism (SNP) analysis

**Figure 8:**
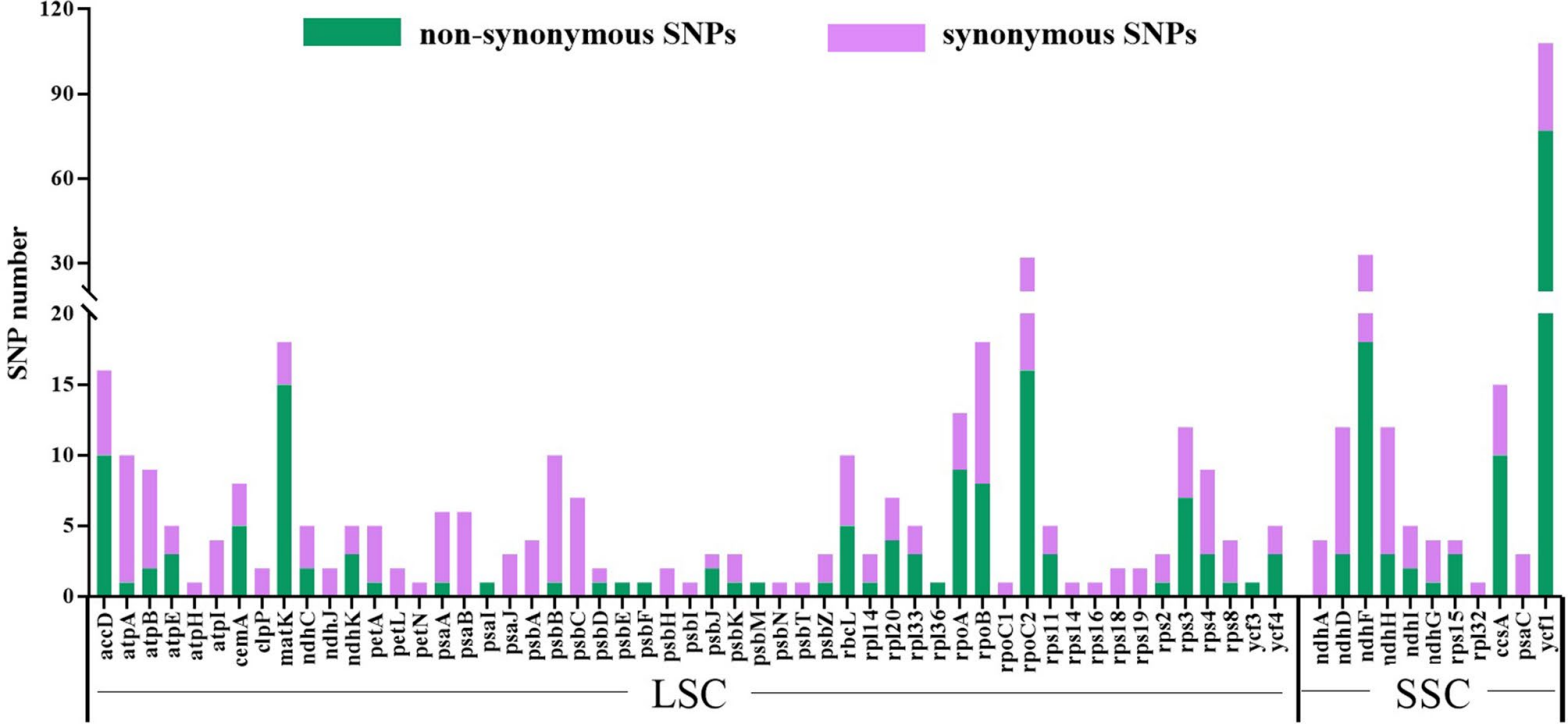
Variation analysis between *S. tonkinensis* and *S. alopecuroides* or *S. flavescens.* Green coloured bars indicate non-synonymous SNPs and purple coloured bars indicate synonymous SNPs.

SNP loci are very useful resources for phylogenetic analysis and species identification^17^. To determine the differences between *S. tonkinensis* and the two other *Sophora* species *S. alopecuroides* and *S. flavescens* at the chloroplast genome level, SNP analysis was carried out with the chloroplast genome of *S. tonkinensis* as the reference sequence. The results revealed 805 SNPs were found in the intergenic region, and 485 SNPs, including 236 non-synonymous SNPs and 249 synonymous SNPs were identified in 64 protein-coding genes. Of these genes, *ycf1* contained the most SNP sites (Fig. 8).

### Phylogenetic analysis

**Figure 9:**
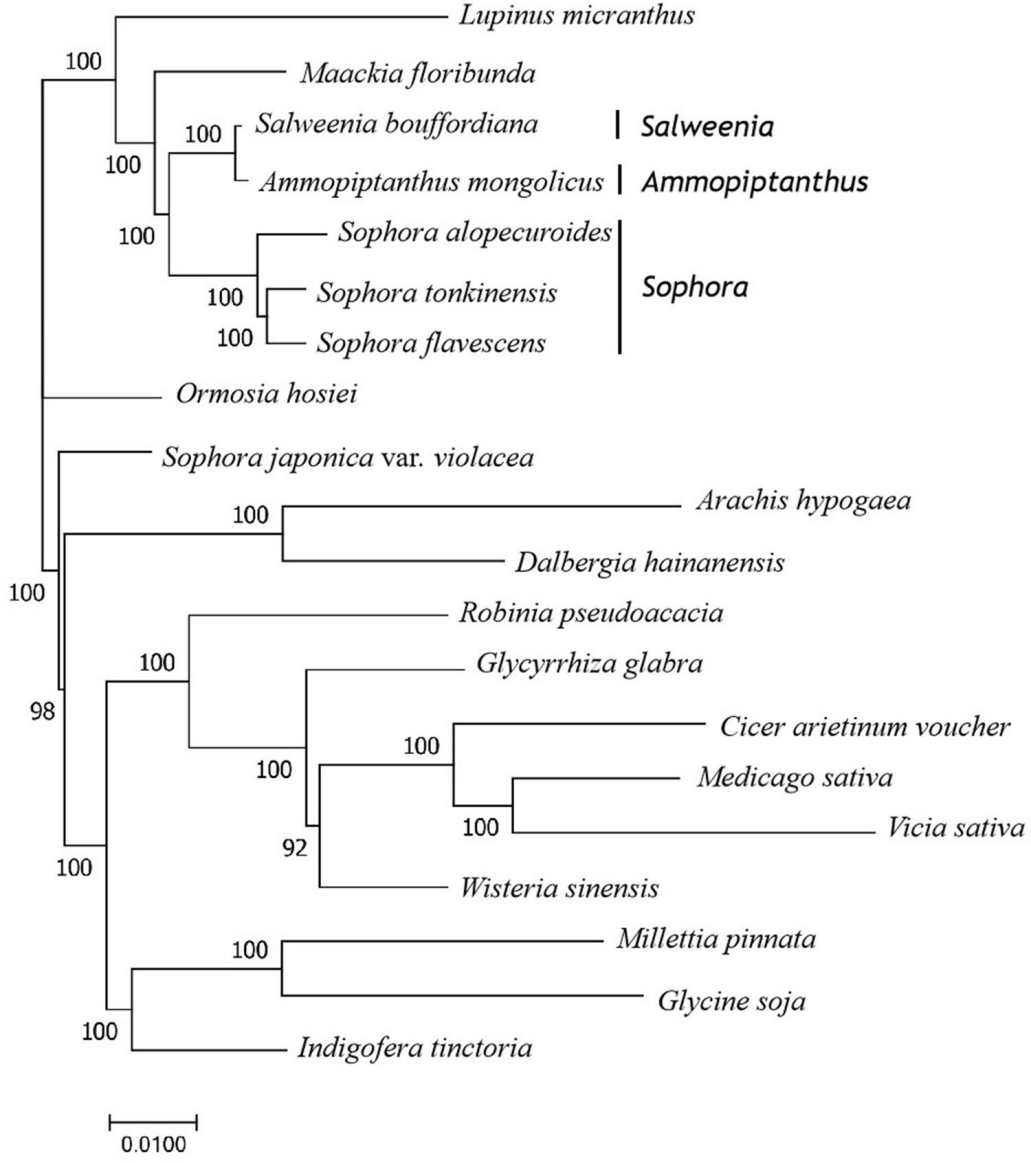
Phylogenetic tree of *S. tonkinensis* and 19 species of subfamily Papilionoideae using Maximum likelihood based on the complete chloroplast genomes. The bootstrap value based on 1,000 replicates is shown on each node.

In the present study, we aligned 20 complete chloroplast genomes of Papilionoideae to reveal the phylogenetic position of *S. tonkinensis* (Fig. 9). The phylogenetic positions of these 20 chloroplast genomes were successfully resolved with full bootstrap support across almost all nodes. We found that *S. tonkinensis* was grouped into *Sophora* with *S. flavescens* and *S. alopecuroides* and *S. tonkinensis* exhibited the closest relationship with *S. flavescens*. A close relationship among the genera *Sophora*, *Salweenia* and *Ammopiptanthus* was also uncovered.

## Discussion

Since the first sequenced plant chloroplast genome was isolated from tobacco^18^, thousands of chloroplast genomes from various species have been sequenced. As of 2019, more than 3,300 chloroplast genome sequences had been recorded in the National Center for Biotechnology Information (NCBI) database. In recent years, DNA barcoding has become a powerful tool for species identification. In plants, commonly used DNA barcodes include the chloroplast genes *rbcL*, *matK* and *psba-trnh* and nuclear genes *ITS* and *ITS2*^19^. Of these, *ITS2* has been suggested as a universal DNA barcode for medicinal plants due to its strong identification ability^12^. However, DNA barcodes do not have enough variation information for species identification of some medicinal plants, such as *Epimedium* and *Fritillariae*^20,21^. Compared with a standardized piece of DNA sequence, the whole chloroplast genome contains more mutation sites and is more efficient in identification. The whole chloroplast genome is used as a superbarcode and has been successfully applied in species identification of some medicinal plants and their closely related species. Cui et al. found that three medicinal and edible *Amomum* taxa (*A. villosum*, *A. villosum* var. *xanthioides* and *A. longiligulare*) could be accurately identified using their whole chloroplast genomes^22^. Chen et al. discovered that the complete chloroplast genome can be used as a superbarcode to identify six *Ligularia* species^23^. The chloroplast genome could distinguish *C. indicum* from its closely related species and might become a potential superbarcode for the identification of these species^24^. Zhu et al*.* found that the complete plastome sequence dataset had the highest discriminatory power for *D. officinale* and its closely related species, indicating that complete plastome sequences can be used to accurately authenticate Dendrobium species^25^. The whole chloroplast genome of *S. tonkinensis* and its hypervariable region, including the most divergent regions (*ycf1*, *ndhF*, *accD*, and *rpoC2*), which are also the genes containing the most SNP sites, and the six positively selected genes (*matK*, *psbE*, *psbF*, *psbM*, *psaI*, and *rpl36*) could be selected as potential DNA barcodes for identification of species in future studies.

Genetic variation plays an important role in the ability of plants to maintain their evolutionary potential to adapt to the ever-changing environment, therefore the maintenance of genetic variation is the main goal of the conservation strategies for most endangered species^26^. SSRs, also known as microsatellites, have high polymorphism rate at the species level^27–30^. Therefore, they have been widely used as effective molecular markers in population genetic and evolution studies^31,32^. Yang et al*.* used eight SSR primer to assess the genetic diversity and structure of 22 natural populations of the endangered medicinal plant *Phellodendron amurense* in China, and proposed proper conservation measures for this species^33^. An ex situ conservation measure for conserving genetically distant populations to maximize the genetic diversity of *Eucommia ulmoides* is recommended based on the genetic analysis diversity within and among the semi-wild and cultivated populations of *E. ulmoides* using two cpSSR loci^34^. In the *S. tonkinensis* chloroplast genome, five types of SSRs (mono-, di-, tri-, tetra-, and penta-nucleotide repeats) and a total of 150 SSR loci with a length of at least 10 bp were identified (Table S4). The mononucleotide repeats were the most abundant SSR. Most of the mononucleotide and dinucleotides are composed of multiple copies of A/T and AT/TA repeats, respectively, this result is similar to that of previous study on *S. alopecuroides*^35^. These SSRs of the *S. tonkinensis* chloroplast genome could be useful biomarkers for genetic diversity studies of wild populations of *S. tonkinensis*, which will help to formulate effective conservation and management strategies for this important medicinal plant.

## Conclusions

In conclusion, the chloroplast genome of *S. tonkinensis* was sequenced on the Illumina HiSeq 2000 platform in this study. SSRs and tandem repeats were identified and 1,760 SSRs were found, most of which were mononucleotides, in the chloroplast genome of *S. tonkinensis*. SSR analysis can provide valuable information for developing highly variable DNA markers for population genetic surveys and other ecological and evolutionary studies of *S. tonkinensis*. Further, we performed phylogenetic analysis of 20 chloroplast genomes and collinearity analysis of three closely related species of *S. tonkinensis*. The contraction and expansion of the IR regions of the three closely related species were also compared. The results of the above analyses provide valuable reference information that will help formulate effective conservation and management strategies as well as molecular identification approaches for this important medicinal plant.

## Materials and methods

### Sample preparation and DNA extraction

*Sophora tonkinensis* leaves were obtained from 2-year-old plants in the Guangxi Botanical Garden of Medicinal Plants (Nanning, China). Genomic DNA was extracted using the DNeasy Plant Mini Kit (Qiagen, Valencia, CA, USA) according to the manufacturer’s instructions.

### Genome assembly and annotation

DNA was randomly fragmented by a Covaris M220 apparatus. After adding the poly “A” tail, the DNA fragments with desired lengths (400–500 bp) were ligated to adapters and purified using the TruSeq™ DNA Sample Prep Kit for Illumina MiSeq sequencing. Before assembly, raw reads were filtered, and the reads with adapters, reads containing too many uncalled bases (“N” characters, ≥ 10%), the reads showing a quality score below 20 (Q < 20), and the duplicated sequences were removed. The optimized sequence was first assembled by using SOAP de Novo v2.04 software (https://soap.genomics.org.cn/)^36^. Second, GapCloser v1.12 software was used to fill the gaps in the assembly results and for base correction. Annotation of the chloroplast genome was conducted using Dual Organellar GenoMe Annotator (DOGMA) software (https://dogma.ccbb.utexas.edu/)^37^, and artificial correction was carried out to predict the genes, rRNAs, and tRNAs in the genome. A circular chloroplast genome map was drawn using the OGDRAW program (https://chlorobox.mpimp-golm.mpg.de/OGDraw.html)^38^.

### Codon usage analysis

RSCU (Relative Synonymous Codon Usage) was computed from the protein-coding gene sequences of the *S. tonkinensis* cp genome. The online program CodonW 1.4.2 (https://codonw.sourceforge.net/) was employed for RSCU and codon frequency analysis^39^.

### Analysis of simple sequence repeats (SSRs) and tandem repeats

The SSRs in the complete chloroplast genome of *S. tonkinensis* were detected using MIcroSAtellite identification tool (MISA) software (https://pgrc.ipk-gatersleben.de/misa/)^40^. The repeat sequences with repeating units of 5, 5, 3, 3, and 3 bp were considered as SSRs for mononucleotide, dinucleotides, trinucleotides, tetranucleotides, and pentanucleotide, respectively. Tandem Repeats Finder (TRF) v4.04 was used to identify tandem repeats^41^.

### Comparative genomic analysis of the *S. tonkinensis* chloroplast genome

The complete chloroplast genome of *S. tonkinensis* was compared with that of three other Papilionoideae species, namely, *S. alopecuroides* [MH_748034], *A. mongolicus* [NC_034742], and *M. floribunda* [KX_388160], in the NCBI plastid database using the mVISTA program with a shuffle-LAGAN model^42^. *S. tonkinensis* was set as the reference.

### Synonymous (K_S_) and non-synonymous (K_A_) substitution rate analysis

The chloroplast genome sequence of *S. tonkinensis* was compared with those of *S. alopecuroides* [MH_748034] and *S. flavescens* [MK_114100] in the NCBI plastid database. The same functional protein-coding exons were extracted and aligned separately to analyse the K_A_ and K_S_ substitution rates using Geneious v7.1.9 software. The aligned sequences were translated into protein sequences and then analysed. The K_A_ and K_S_ substitution rates for each protein-coding exon were calculated in DnaSP^43^. The ratios K_A_/K_S_ > 1, K_A_/K_S_ = 1 and K_A_/K_S_ < 1 indicate positive selection, neutral selection and negative selection, respectively^44^.

### Phylogenetic analysis

The phylogenetic analysis was based on the complete chloroplast genome sequence of *S. tonkinensis* and 19 species of Papilionoideae downloaded from the NCBI database (https://www.ncbi.nlm.nih.gov/), including two species of *Sophora* species, *S. alopecuroides* and *S. flavescens*, and 17 chloroplast genomes from the other basic lineage of Papilionoideae. The maximum likehood method was used to infer the phylogenetic relationship with 1,000 bootstrap replicates in MEGA 5.0^45^.

## Supplementary information

Supplementary Information 1.
