# Supplementary material for: The complete chloroplast genome sequence of the medicinal plant Sophora tonkinensis
Source: Sci Rep. 2020 Jul 27;10:12473. doi: 10.1038/s41598-020-69549-z (PMC7385175; doi:10.1038/s41598-020-69549-z)
Supplement: Supplementary file 1 — Supplementary Information 1. [file 41598_2020_69549_MOESM1_ESM.docx]

**The complete chloroplast genome sequence of the medicinal plant *Sophora tonkinensis***

**Fan Wei^1^, Danfeng Tang^1^, Kunhua Wei^1^, Fang Qin ^1^, Linxuan Li^1^, Yang Lin^1^, Yanxia Zhu^1^, Aziz Khan^2^, Muhammad Haneef Kashif^2^, Jianhua Miao^1*^**

^1^ Guangxi Key Laboratory of Medicinal Resources Protection and Genetic Improvement, Guangxi Botanical Garden of Medicinal Plants, Nanning, Guangxi, 530023, China

^2^ Key Laboratory of Plant Genetics and Breeding, College of Agriculture, Guangxi University, Nanning, Guangxi, 530005, China

*Corresponding author: mjh1962@vip.163.com; Tel: +86-186-7719-1399

| **Gene** | **Location** | **Exon I** | **Intron I** | **Exon II** | **Intron II** | **Exon II** |
| --- | --- | --- | --- | --- | --- | --- |
|  |  | **Nucleotides in base pairs** | | | | |
| *rpoC1* | LSC | 18004 | 18436 | 19206 |  |  |
| *atpF* | LSC | 28033 | 28184 | 28943 |  |  |
| *ycf3* | LSC | 51750 | 51903 | 52635 | 52865 | 53618 |
| *rps16* | LSC | 57081 | 57313 | 56126 |  |  |
| *clpP* | LSC | 70084 | 70312 | 70986 | 71283 | 72158 |
| *petB* | LSC | 75993 | 76635 | 75223 |  |  |
| *petD* | LSC | 77536 | 78011 | 76807 |  |  |
| *rpl16* | LSC | 81919 | 82318 | 83416 |  |  |
| *rpl2* | LSC | 85165 | 85602 | 86305 |  |  |
| *ndhB* | IR | 95803 | 96559 | 97244 |  |  |
| *ndhA* | SSC | 120233 | 120775 | 121956 |  |  |
| *ndhB* | IR | 142488 | 143211 | 143896 |  |  |

**Table S1.** Locations and lengths of intron-containing genes in the *S. tonkinensis* chloroplast genome.

| **SSR type** | **SSR sequence** | **Size (bp)** | **Start** | **End** | **Location** |
| --- | --- | --- | --- | --- | --- |
| Tetra | (AATG)3 | 12 | 35,177 | 35,188 | *trnT-*UGU*/trnL-*UAA (IGS) |
| Tetra | (TTAA)3 | 12 | 66,082 | 66,093 | *petG/trnW-*CCA (IGS)(IGS) |
| Tetra | (ATAA)3 | 12 | 68,049 | 68,060 | *rpl33/rpl18* (IGS) |
| Tetra | (ATGT)3 | 12 | 78,339 | 78,350 | *rpoA* (CDS) |
| Tetra | (TTCA)3 | 12 | 1,738 | 1,759 | *psbA/matK* (IGS) |
| Tetra | (AAAT)3 | 15 | 66,830 | 66,844 | *petG/psaJ* (IGS) |
| Penta | (TATAG)3 | 15 | 2,010 | 2,024 | *psbA/matK* (IGS) |
| Penta | (TAAGA)3 | 15 | 27,470 | 27,484 | *atpI/atpH* (IGS) |
| Penta | (ATGAT)3 | 15 | 39,692 | 39,706 | *trnE-*UUG*/trnT-*GGU (IGS) |
| Penta | (ATATG)3 | 15 | 71,836 | 71,850 | *clpP/psbB* (IGS) |
| Penta | (TTCTA)3 | 15 | 114,726 | 114,740 | *trnL-UAG/ccsA* (IGS) |
| Penta | (TTCTT)3 | 15 | 126,798 | 126,817 | *ycf1* (CDS) |

**Table S2.** Distribution of tetra and penta SSRs in the *S. tonkinensis* chloroplast genome.

| **No.** | **Repeat length (bp)** | **Consensus size × copy number** | **Start** | **End** | **Location** |
| --- | --- | --- | --- | --- | --- |
| 1 | 68 | 26 × 2.6 | 4,677 | 4,744 | *trnK-*UUU*/rbcL* (IGS) |
| 2 | 43 | 21 × 2.0 | 10,648 | 10,690 | *trnV-*UAC*/ndhC* (IGS) |
| 3 | 42 | 21 × 2.0 | 10,841 | 10,882 | *trnV-*UAC*/ndhC* (IGS) |
| 4 | 307 | 152 × 2.0 | 25,121 | 25,427 | *rpoC2/rps2* (IGS) |
| 5 | 258 | 128 ×2.0 | 30,917 | 31,174 | *atpA/trnR-*UCU (IGS) |
| 6 | 536 | 268 ×2.0 | 37,706 | 38,241 | *petN/psbM* (IGS) |
| 7 | 132 | 63 × 2.1 | 40,049 | 40,180 | *trnE-*UUC*/trnT-*GGU (IGS) |
| 8 | 47 | 19 × 2.5 | 40,616 | 40,662 | *trnT-*GGU*/psbD* (IGS) |
| 9 | 45 | 19 × 2.4 | 45,458 | 45,502 | *psbZ/trnG-*GCC (IGS) |
| 10 | 50 | 25 × 2.0 | 51,422 | 51,471 | *psaA/ycf3* (IGS) |
| 11 | 45 | 22 × 2.0 | 55,168 | 55,212 | *psbK/trnQ-*UUG (IGS) |
| 12 | 104 | 51 × 2.0 | 55,747 | 55,850 | *trnQ-*UUG*/rps16* (IGS) |
| 13 | 52 | 26 × 2.0 | 71,489 | 71,540 | *clpP* (intron) |
| 14 | 45 | 19 × 2.4 | 84,754 | 84,798 | *rps3/rps19* (IGS) |
| 15 | 65 | 21 × 3.1 | 88,953 | 89,017 | *ycf2* (CDS) |
| 16 | 70 | 35 × 2.0 | 110,562 | 110,631 | *trnN-*GUU*/ycf1* (IGS) |
| 17 | 60 | 32 × 1.9 | 111,102 | 111,161 | *NdhF* (CDS) |
| 18 | 145 | 68 × 2.1 | 113,589 | 113,733 | *ndhF/rpl32* (IGS) |
| 19 | 60 | 31 × 2.0 | 114,357 | 114,416 | *rpl32/trnL-*UAG (IGS) |
| 20 | 45 | 22 × 2.0 | 115,843 | 115,887 | *ccsA/ndhD* (IGS) |
| 21 | 139 | 68 × 2.0 | 123,813 | 123,951 | *ndhH/rps15* (IGS) |
| 22 | 73 | 36 × 2.0 | 129,963 | 130,035 | *ycf1/trnN-*GUU (IGS) |
| 23 | 65 | 21 × 3.1 | 151,437 | 151,501 | *ycf2* (CDS) |

**Table S3.** Distribution of tandem repeats in the *S. tonkinensis* chloroplast genome. IGS: Intergenic spacer; CDS: protein-coding region.

| **Repeats** | **3** | **4** | **5** | **6** | **7** | **8** | **9** | **10** | **11** | **12** | **13** | **14** | **15** | **16** | **17** | **18** | **19** | **20** | **21** | **Total** |
| --- | --- | --- | --- | --- | --- | --- | --- | --- | --- | --- | --- | --- | --- | --- | --- | --- | --- | --- | --- | --- |
| A/T |  |  |  |  |  |  |  | 34 | 23 | 18 | 10 | 12 | 7 |  | 2 |  | 1 |  |  | 107 |
| C/G |  |  |  |  |  |  |  | 5 | 1 | 1 |  |  |  |  |  |  |  |  | 1 | 8 |
| AG/CT |  |  | 2 |  |  |  |  |  |  |  |  |  |  |  |  |  |  |  |  | 2 |
| AT/TA |  |  | 7 | 10 | 3 | 3 | 4 | 1 |  |  |  |  |  |  |  |  |  |  |  | 28 |
| AAG/CTT |  | 1 |  |  |  |  |  |  |  |  |  |  |  |  |  |  |  |  |  | 1 |
| AAT/ATT |  | 2 |  |  |  |  |  |  |  |  |  |  |  |  |  |  |  |  |  | 2 |
| ATATC/ATATG | 2 |  |  |  |  |  |  |  |  |  |  |  |  |  |  |  |  |  |  | 2 |
| **Total** |  |  |  |  |  |  |  |  |  |  |  |  |  |  |  |  |  |  |  | **150** |

**Table S4.** Frequency of classified repeat types with a length of at least 10 bp (considering sequence complementary) in the *S. tonkinensis* chloroplast genome.
